# Clinical Management of Embryonal Tumor with Multilayered Rosettes: The CCMC Experience

**DOI:** 10.3390/children9101560

**Published:** 2022-10-14

**Authors:** Zengyan Li, Zhiming Wu, Youhong Dong, Dongdong Zhang

**Affiliations:** 1 Department of Oncology, Xiangyang No. 1 People’s Hospital, Hubei University of Medicine, Xiangyang 441000, China; 2 Department of Orthopedics, Xiangyang No. 1 People’s Hospital, Hubei University of Medicine, Xiangyang 441000, China; 3 Xinhua Hospital Affiliated to Shanghai Jiao Tong University School of Medicine, Shanghai 200092, China

**Keywords:** embryonal tumor with multilayered rosettes, *C19MC*, surgery, adjuvant chemotherapy, radiotherapy

## Abstract

Objective: Embryonal tumors with multilayered rosettes (ETMRs) are highly aggressive pediatric brain tumors with poor prognosis. No standard treatment strategy for them exists because of their rarity. This study aimed to share experiences on the clinical diagnosis and treatment of ETMRs at China Children’s Medical Center (CCMC). Methods: Patients who received a diagnosis of an ETMR between January 2017 and June 2020 were included. Clinical characteristics, such as age of onset, tumor size, stage, tumor site, treatment strategy, and clinical outcome, were retrospectively analyzed. Results: There were four boys and one girl within 4 years who received a diagnosis during this 4-year timeframe, and were thus included. The average age of morbidity was 29 months (range 16–66 months). The common clinical presentation was headaches and nausea caused by intracranial hypertension. All four patients were chromosome 19 microRNA cluster (*C19MC*) amplification positive. Two patients achieved complete remission, and one patient attained partial remission after multimodal treatment. Of the two deaths, one died from the rapid progression of the disease and another from tumor-related complications. Conclusion: ETMRs are extremely rare brain tumors with a high, early mortality in children. Surgery is the mainstream treatment for ETMRs. Some patients may also benefit from postoperative adjuvant chemotherapy and radiotherapy.

## 1. Introduction

Embryonal tumors with multilayered rosettes (ETMRs) are rare and highly aggressive brain tumors that primarily affect infants and young children. The term “ETMR” was first described in the 2016 World Health Organization (WHO) classification of central nervous system (CNS) tumors [1]. ETMRs were historically classified as ependymoblastoma, medulloepithelioma, and embryonal tumor with abundant neuropil and true rosettes (ETANTR). Some ETMRs were also previously diagnosed as a subtype of CNS primitive neuroectodermal tumor because of the specific histologic presentation [2]. The characteristic histology of ETMRs is the presence of undifferentiated neuroepithelial cells forming multilayered rosettes. Furthermore, amplification of the chromosome 19 microRNA cluster (*C19MC*) at 19q13.41–42 and the overexpression of the RNA binding protein Lin28A were the typical molecular genetic features for ETMRs, present in approximately 90% of cases [3]. Based on these sensitive and specific markers, the updated 2021 WHO classification of CNS tumors classified ETMRs into the common *C19MC* type and DICER alteration type according to their genetic subtypes [4]. ETMRs usually occur in the cerebral hemispheres, although they can also originate in the brain stem and cerebellum. Not only can ETMRs develop leptomeningeal dissemination, but they can also present with extracranial invasive growth and metastases. The most common clinical presentation is intracranial hypertension as a result of the large and solid intracranial masses at the time of neuroimaging examination.

The incidence of ETMRs is extremely low; fewer than 300 cases had been reported by the end of December 2020 [5]. The recent multicenter clinical study reported the incidence in Europe to be 1.35/1001,000,000 (aged 1–4 years) [6]. In the last two decades, despite the use of multidisciplinary treatments, such as maximal safe resection, high-dose chemotherapy, radiotherapy, and autologous bone marrow transplantation (ABMT), the prognosis of ETMRs has remained dismal. The reported median survival time is twelve months, and the 5-year event-free survival rate is still lower than 30% [7]. There are no standard treatment regimens for this malignancy due to the paucity of cases. We share our experience on ETMRs and the treatment strategy formulated by China Children’s Medical Center (CCMC) in this study.

## 2. Patients and Methods

### 2.1. Patients

Patients with a newly diagnosed ETMR and who were treated in the Pediatric Cancer Center of Xiangyang No.1 People’s Hospital affiliated with Hubei University of Medicine between January 2017 and June 2020 were included. The diagnostic evaluation of an ETMR was performed by histological and immunohistochemical analysis and the status of *C19MC* according to the 2016 revision of the WHO classification of CNS tumors. The amplification of *C19MC* was detected by fluorescence in situ hybridization. The electronic medical records were carefully reviewed. This study was approved by the Ethics and Scientific Committee of Hubei University of Medicine with Approval Number XYY2021002. Parents gave written informed consent before the therapy was given.

### 2.2. Treatment

All patients underwent surgical resection and pathological examination. Five cycles of chemotherapy were performed after surgery. The CHEMO regimen, which combined cyclophosphamide, vincristine, etoposide, cisplatin, and a high dose of methotrexate, was used as the adjuvant chemotherapy in our study. For patients below 18 months at the time of indication for chemotherapy, the doses of chemotherapeutic drugs were adjusted according to the weight, other than the body surface area. Generally, cisplatin was given for 5 courses on Day 1, at a dose of 105 mg/m^2^ for patients over 18 months (3.5 mg/kg for patients below 18 months); vincristine was given for the first three course on Days 1, 8, and 15, at a dose of 1.5 mg/m^2^ (or 0.05 mg/kg); etoposide was given for 5 courses on Days 2 and 3, at a dose of 120 mg/m^2^ (or 4.0 mg/kg); cyclophosphamide was given for 5 courses on Days 2 and 3, at a dose of 2000 mg/m^2^ (or 65 mg/kg); high-dose methotrexate was given for 5 courses on Day 4, at a dose of 12.0 g/m^2^ (or 400 mg/kg). The detailed information is shown in Table 1. For patients below 36 months, irradiation was not recommended. For patients over 36 months, the addition of radiotherapy was determined according to the extent of resection margin, age, and performance status. The prescribed craniospinal dose was 23.4 Gy, with daily fractions of 1.8 Gy, followed by a boost up to 54.0 Gy to the tumor region.

### 2.3. Response and Toxicity

The surgical resection can be classified into complete tumor resection (CTR), gross total resection (GTR), and subtotal resection (STR). The classification was determined by postoperative magnetic resonance imaging. The Response Assessment in Neuro-Oncology (RANO) criteria were used to assess treatment response [8]. Complete remission (CR) was defined as the absence of any residual radiologic abnormalities. Partial remission (PR) was defined as a reduction of tumor volume ≥ 50%. Progressive disease (PD) was defined as an increase of tumor volume ≥ 25%, the appearance of new lesions, or distant metastases.

The routine examination, including hematological and biochemistry tests and electrocardiograph, was performed before and after every cycle of chemotherapy. Treatment-related adverse effects were graded according to the National Cancer Institute Common Terminology Criteria for Adverse Events version 4.0 [9].

## 3. Result

### 3.1. Patients

A total of 342 patients with CNS tumor were admitted to our center between 2017 and 2022, and only 5 children were diagnosed with an ETMR, including four boys and one girl. Among the five children, four children were *C19MC* amplification positive, only one was *C19MC* amplification negative, and the presence of DICER1 alteration was not assessed. The onset age ranged from 16–66 months. The supratentorial and infra-tentorial regions were the most common tumor sites. The tumor volume in most cases was larger than 5.0 cm × 5.0 cm × 3.0 cm. The detailed clinical data for all five children are shown in Table 2.

### 3.2. Treatment and Toxicity

All of the children received surgery followed by adjuvant chemotherapy. Among all of the patients, there were two patients with R0 resection and two patients with R1 resection. Only one patient received R2 resection, due to tumor invasion of the medulla oblongata; this patient received whole-brain and spinal cord radiotherapy after five cycles of the CHEMO regimen.

Common adverse reactions of the treatment were myelosuppression and gastrointestinal reaction. All of the children showed grade III–IV myelosuppression after high-intensity chemotherapy. However, rapid recovery can be achieved after blood component transfusion and the injection of recombinant human granulocyte stimulating factor. The patient who received R2 resection completed chemotherapy and radiotherapy successfully without any other side effects except for mild hearing loss.

### 3.3. Treatment Response and Clinical Outcome

After the treatment of adjuvant chemotherapy, two patients achieved continuous CR, one patient achieved PR, and two patients achieved PD. By the end of the follow-up time, the two children with PD died due to the disease’s progression. Patient 5 died from the rapid progression of disease after two cycles of the CHEMO regimen. Patient 4 died of severe infection and multiple organ failure eighteen months later. The remaining three patients are still alive, including the patient who received R2 resection and radiotherapy.

## 4. Discussion

ETMRs primarily occur in children under four years of age; the median onset age is 26–29 months. Many patients die within one year of the diagnosis, and the 5-year overall survival rate is lower than 30% [7]. Therefore, more effective treatment strategies are required.

The clinical presentation of ETMRs is similar to other brain tumors, depending on the tumor site and size and the presence or absence of cerebrospinal fluid circulation obstruction [10]. ETMRs usually occur in the supratentorial region, although they can also occur in the brainstem and cerebellum [11]. The ETMR usually metastasizes locally. However, 18.9% of patients exhibit distant metastases at the time of diagnosis [12]. The most common clinical manifestations of ETMRs are nausea, vomiting, and headache caused by increased intracranial pressure. In addition, some children may also show paralysis, epilepsy, visual impairment, ataxia, and torticollis [10]. In our center, all of the children presented clinical symptoms related to increased intracranial pressure, and two of the patients presented with balance problems due to tumor lesions located in the fourth ventricle.

ETMRs may be misdiagnosed as medulloblastoma, ependymoma, atypical teratoid/rhabdomyoma (AT/RT), and other types of malignant brain tumors [11]. Approximately 95% of ETMRs have *C19MC* amplification, whereas *C19MC* is not overexpressed in the other brain tumors listed above. Moreover, the Lin28A protein was overexpressed in the multilayered rosettes area and the poorly differentiated tumor area in the ETMR tissue. Previous studies suggested that Lin28A and *C19MC* can be used as specific diagnostic markers for ETMRs [11]. However, it should be noted that there are still 5% of children with an ETMR who do not have the *C19MC* amplification, and most of these children have the DICER1 mutation [12]. Furthermore, the overexpression of Lin28A also could be detected in 25% of AT/RT children. Therefore, we suggest that histopathological examination, *C19MC* and DICER1 status, and Lin28A protein detection should be performed for the diagnosis of an ETMR.

Surgery is the cornerstone treatment for ETMRs. It can directly relieve the clinical symptoms of the disease. Moreover, the pathological and molecular biological examination of specimens obtained via surgery are key to confirming the diagnosis. In some cases, complete resection of a tumor cannot be guaranteed, especially those that are located in or near the brainstem. These patients require careful preoperative evaluation to determine whether gross total resection, or subtotal resection, could be performed. Complete resection can provide patients with better survival benefits [13]. The location of tumor incidence and the absence of distant metastases during initial treatment are also closely related to the prognosis of the disease. It has been reported that tumors located in the supratentorial region have a good prognosis; this is probably because most tumors located in the supratentorial region can be completely resected.

It is still controversial whether ETMR patients should receive adjuvant chemotherapy after surgery. A retrospective study from France suggested that although postoperative chemotherapy did not prolong long-term survival, high-intensity chemotherapy could reduce the prescribed dose of whole-brain and spinal cord radiotherapy and reduce the long-term toxicity of radiotherapy [14]. A prospective European study in 2021 suggested that high-dose chemotherapy could improve the overall survival rate [6]. Therefore, we think adjuvant chemotherapy is important for patients with an ETMR.

So far, the PNET-HR protocol, German HIT protocol, COG study, and Head Start regimen are widely used for the treatment of ETMRs [15]. What these regimens have in common is using cyclophosphamide, etoposide, cisplatin, and methotrexate as the basis of therapy. Based on the Head Start series regimen [13], we formulated the CHEMO regimen, which combines cyclophosphamide, vincristine, etoposide, cisplatin, and high-dose methotrexate. We increased the dosage of cyclophosphamide and methotrexate to ensure a sufficient amount of the drug delivered to the tumor region. This regimen was generally tolerated and could be used as postoperative adjuvant chemotherapy for ETMRs.

The previous studies indicated that postoperative adjuvant radiotherapy can improve the prognosis and prolong the overall survival rate for ETMR patients [16]. A retrospective analysis suggested that a small portion of children with an ETMR could achieve long-term survival when they received whole-brain and spinal cord radiotherapy [17]. In our study, only one patient received whole-brain and spinal cord radiotherapy after R2 resection and adjuvant chemotherapy; this patient was still alive after 24 months of follow-up. This result suggested that patients with subtotal resection may benefit from radiotherapy, which is also consistent with the most recent data from the Canadian Society for Rare Brain Tumors in 2021 [18]. However, the European P-HIT clinical trial showed that children who can achieve complete remission after adjuvant chemotherapy had a longer disease-free survival, with or without radiation [6]. ETMRs primarily affect very young children. Radiotherapy can lead to skull deformity and cognitive impairment. Furthermore, radiation can also lead to developmental abnormalities and even the occurrence of second tumors in the long term. Therefore, clinicians must carefully weigh benefits and risks.

**Figure 1:**
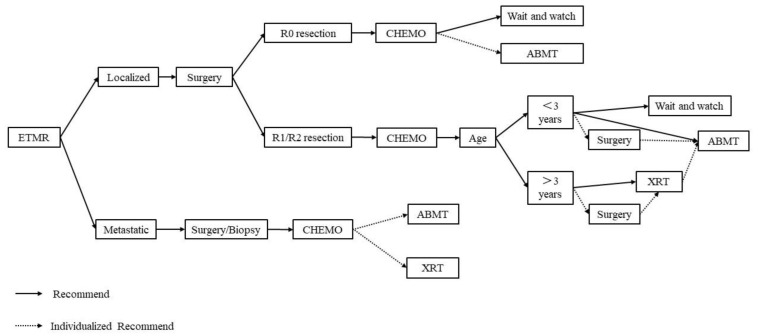
The treatment strategy for ETMR.

Based on the results from previous studies and the treatment experience from our center, we formulated a treatment strategy for ETMRs (Figure 1). Patients with R0 resection can be followed up by observation or ABMT after the completion of adjuvant chemotherapy. For patients with R1 or R2 resection, without distant metastases at initial diagnosis and who achieved CR or PR after postoperative chemotherapy, whether radiation was received depended on the age. Generally, radiation was recommended for patients over 3 years old. For patients with distant metastases at initial diagnosis, surgery combined with adjuvant chemotherapy is the main treatment. Those patients had a poor Eastern Cooperative Oncology Group (ECOG) performance status and prognosis, and whether radiotherapy should be given should be determined on an individual basis [6,18].

It is urgent to develop new treatment methods to improve the overall prognosis of ETMRs. It has been reported that amplification of *C19MC* can lead to the upregulation of DNA methyltransferase, DTMN3B, in tumors of children with an ETMR; DTMN3B is crucial for the development of neural tubes in the early stage of an ETMR, suggesting that we can treat ETMRs by using epigenetic drugs [19]. Some other chemotherapeutic drug combinations, such as topoisomerase inhibitors combined with anthracyclines and actinomycin, or some other tumor targeting regimens, such as AT/RT and rhabdomyosarcoma, have also shown promising results in ETMRs [20,21]. In addition, some clinical trials are developing emerging targets for drugs, such as a POLO-like kinase inhibitor (volasertib), an Aurora kinase A inhibitor alisertib, a mammalian mTOR inhibitor (MLN0128), and a Sonic hedgehog inhibitor (arsenic trioxide), which have shown some therapeutic potential for ETMRs [21,22]. Therefore, the ETMR treatment exploration road is full of thorns, but the future is promising.

In conclusion, we formulated a treatment strategy for ETMRs, varied according to the tumor stage. Maximum excision is the mainstream treatment, but patients could also benefit from adjuvant chemotherapy. Whether radiation should be given depends on the age and ECOG performance status. Finally, small molecular inhibitors may be promising agents for ETMR, but further studies are needed.

## Figures and Tables

**Table 1:** The detailed information for the CHEMO regimen.

Drug	Dose	Day
Cisplatin (CDDP)	105 mg/m^2^	D1
Vincristine (VCR)	1.5 mg/m^2^ (Dmax = 2 mg)	D1, 8, 15 (The first three cycles)
Etoposide (VP-16)	120 mg/m^2^	D2, 3
Cyclophosphamide (CTX)	2000 mg/m^2^	D2, 3
High-dose methotrexate (HD-MTX)	12 g/m^2^	D4

Note: For patients below 18 months, CDDP was given at a dose of 3.5 mg/kg; VCR was given at a dose of 0.05 mg/kg; VP-16 was given at a dose of 4.0 mg/kg; CTX was given at a dose of 65 mg/kg; HD-MTX was given at a dose of 400 mg/kg. The CHEMO regimen was given at the interval of 21 days.

**Table 2:** Clinical characteristics of children with an ETMR.

Patient	Sex	Age (Months)	Tumor Site	Clinical Symptoms	Tumor Size (cm^3^)	*C19MC* Amplification	Resection	Cycles of Chemotherapy	Radiotherapy	Myelosuppression	Response	Outcome (Months)
1	Female	17	The frontal temporal lobe	Limb weakness, vomiting	7.3 × 7.2 × 6.8	Yes	CTR	5	No	III	CR	NED (19)
2	Male	16	The fourth ventricle	Walking instability	3.8 × 3.6 × 5.3	No	CTR	5	No	IV	CR	NED (14)
3	Male	25	The fourth ventricle	Walking instability	6.0 × 6.0 × 3.8	Yes	STR	5	Yes	IV	PR	AWD (24)
4	Male	66	Cavernous sinus and slope	Headache, vomiting	1.6 × 2.7 × 4.2	Yes	GTR	2	No	IV	PD	DOC (18)
5	Male	21	The temporal lobe	Drowsiness, vomiting, convulsions	6.1 × 5.9 × 3.0	Yes	GTR	2	No	IV	PD	DOD (6)

## Data Availability

The clinical data supporting the conclusions of this manuscript will be made available by the authors.
